# A case-control study of tackle based head impact event (HIE) risk factors from the first three seasons of the National Rugby League Women's competition

**DOI:** 10.3389/fspor.2023.1080356

**Published:** 2023-06-02

**Authors:** Shreya McLeod, Ross Tucker, Suzi Edwards, Ben Jones, Georgia Page, Mily Spiegelhalter, Stephen W. West, Grant L. Iverson, Andrew J. Gardner

**Affiliations:** ^1^ School of Medicine and Public Health, College of Health, Medicine, & Wellbeing, The University of Newcastle, Callaghan, NSW, Australia; ^2^ Discipline of Physiotherapy, School of Allied Health, Australian Catholic University, Sydney, NSW, Australia; ^3^ Department of Exercise, Institute of Sport and Exercise Medicine (ISEM), University of Stellenbosch, South Africa; ^4^ World Rugby Ltd., Dublin, Ireland; ^5^ Faculty of Medicine and Health, Sydney School of Health Sciences, Discipline of Exercise and Sport Science, The University of Sydney, Camperdown, NSW, Australia; ^6^ Carnegie Applied Rugby Research (CARR) Centre, Carnegie School of Sport, Leeds Beckett University, Leeds, United Kingdom; ^7^ Division of Physiological Sciences, Department of Human Biology, UCT Research Centre for Health Through Physical Activity (HPALS), Lifestyle and Sport, Faculty of Health Sciences, University of Cape Town, Cape Town, South Africa; ^8^ England Performance Unit, Rugby Football League, Red Hall, Leeds, United Kingdom; ^9^ Leeds Rhinos Rugby League Club, Leeds, United Kingdom; ^10^ Premiership Rugby, London, United Kingdom; ^11^ Centre for Health, and Injury & Illness Prevention in Sport, University of Bath, Bath, United Kingdom; ^12^ UK Collaborating Centre on Injury and Illness Prevention in Sport (UKCCIIS), University of Bath, Bath, United Kingdom; ^13^ Sport Injury Prevention Research Centre, Faculty of Kinesiology, University of Calgary, Calgary, AB, Canada; ^14^ Department of Physical Medicine and Rehabilitation, Harvard Medical School, Boston, MA, United States; ^15^ Department of Physical Medicine and Rehabilitation, Spaulding Rehabilitation Hospital, Charlestown, MA, United States; ^16^ Department of Physical Medicine and Rehabilitation, Schoen Adams Research Institute at Spaulding Rehabilitation, Charlestown, MA, United States; ^17^ MassGeneral Hospital for Children Sports Concussion Program, Boston, MA, United States; ^18^ Hunter Medical Research Institute, New Lambton, NSW, Australia

**Keywords:** head impact events, rugby league, tackle, female athlete, brain concussion, mild traumatic brain injury

## Abstract

**Objective:**

The tackle is the most injurious event in rugby league and carries the greatest risk of concussion. This study aims to replicate previous research conducted in professional men's rugby league by examining the association between selected tackle characteristics and head impact events (HIEs) in women's professional rugby league.

**Methods:**

We reviewed and coded 83 tackles resulting in an HIE and every tackle (6,318 tackles) that did not result in an HIE for three seasons (2018–2020) of the National Rugby League Women's (NRLW) competition. Tackle height, body position of the tackler and ball carrier, as well as the location of head contact with the other player's body were evaluated. Propensity of each situation that caused an HIE was calculated as HIEs per 1,000 tackles.

**Results:**

The propensity for tacklers to sustain an HIE was 6.60 per 1,000 tackles (95% CI: 4.87–8.92), similar to that of the ball carrier (6.13 per 1,000 tackles, 95% CI: 4.48–8.38). The greatest risk of an HIE to either the tackler or ball carrier occurred when head proximity was above the sternum (21.66 per 1,000 tackles, 95% CI: 16.55–28.35). HIEs were most common following impacts between two heads (287.23 HIEs per 1,000 tackles, 95% CI: 196.98–418.84). The lowest propensity for both tackler (2.65 per 1,000 tackles, 95% CI: 0.85–8.20) and ball carrier HIEs (1.77 per 1,000 tackles, 95% CI: 0.44–7.06) occurred when the head was in proximity to the opponent's shoulder and arm. No body position (upright, bent or unbalanced/off feet) was associated with an increased propensity of HIE to either tackler or ball carrier.

**Conclusions:**

In the NRLW competition, tacklers and ball carriers have a similar risk of sustaining an HIE during a tackle, differing from men's NRL players, where tacklers have a higher risk of HIEs. Further studies involving larger samples need to validate these findings. However, our results indicate that injury prevention initiatives in women's rugby league should focus on how the ball carrier engages in contact during the tackle as well as how the tackler executes the tackle.

## 1. Introduction

Rugby league is a full contact, collision sport involving multiple tackle events within a single game (1, 2). In Australia, the highest level of domestic competition for men is the National Rugby League (NRL). Recently, women's domestic club leagues have been created in Australia, with the National Rugby League Women's (NRLW) competition commencing in 2018, and in the United Kingdom, the Women's Super League, beginning in 2017. In women's rugby league, match concussions ranging between 2.3 concussions per 1,000 head contacts and 10.3 concussions per 1,000 match hours have been reported (3, 4). The incidence in professional women's rugby union has been reported as between 5 and 18.6 concussions per 1,000 match hours (5, 6). Since women's rugby league and union are at a relatively early stage, it is unclear whether such differences are the result of inequities in resourcing, access to facilities, skill and conditioning levels of players, opportunities to train and compete (7–9), or relatively immature injury surveillance systems (10). However, in other sports, it has been found that women are more likely to experience concussions, and exhibit greater susceptibility to concussions than men (11–14).

In the men's NRL, the tackle is the game play event associated with the greatest risk and number of concussions, with the tackler more frequently concussed than the ball carrier (1, 15–21). Video technology has been adopted by many professional sports to identify risk factors for head impacts (15, 22–25), providing insights into the mechanisms leading to concussions, which, in turn, may determine which tackle-based interventions might be introduced to reduce risk in rugby league. Head-to-head contact or head contact with a bony body part (e.g., shoulder) are the most common mechanisms for head impact events (HIEs) in concussed tacklers. Upright tackles approximating head-to-head contact result in a greater propensity for head impact events in laboratory evaluations, and video coding of game footage in rugby league and rugby union (17, 26–28). It remains unknown whether these risk factors are also present in women's rugby league. This is of particular interest because recent research has shown that NRLW players engaged in a greater mean number of tackle events per game (*n* = 512) (29) compared to women's rugby union players (*n* = 280) (30), with hookers recording the most tackles per game (*n* = 26.4) (31).

For the purposes of this study, an HIE is defined as a clear head impact sustained by a player in the tackle, reviewed on video footage. This approach was chosen to maximize the number of cases available for analysis, since the Head Injury Assessment (HIA) and confirmed concussion numbers across the three seasons is small. This is, therefore, a preliminary study, the first of its kind to explore mechanisms for head impact events in the NRLW, that may in the future be expanded to examine those head impact events that reach a level of clinical significance, requiring either temporary or permanent removal of player (HIA cases) or diagnosed concussions. The primary aim of the present study was to code and review video footage of tackles resulting in HIEs, including those that resulted in an HIA or a medically diagnosed concussion, in the highest level of Australian women's club rugby league.

## 2. Methods

### 2.1. Participants

This retrospective, case-control video analysis study was conducted in the NRLW competition over three seasons (2018–2020). At the time of analysis, the NRLW was comprised of four teams competing over four rounds. The NRLW was comprised of four teams competing over four rounds (a total of 7 games per season, for a total sample of 21 games) (32). NRLW squads are comprised of marquee, reserve grade, and under 20s players. Over the three seasons, a total of 154 unique players participated in at least one NRLW match. All players, in accordance with the NRL and Rugby League Players Association Collective Bargaining Agreement, consented *a priori* to the collection of their deidentified injury data, for the purposes of research. In addition, the study was approved by The University of Newcastle's Human Research Ethics Committee (H-2012-0344) and conducted in accordance with the standards of ethics outlined in the Declaration of Helsinki.

### 2.2. Procedures

Head impact events were coded via the Stats Edge Platform (edge.stats.com). All tackle events were coded by a single analyst (SM) using a predefined coding matrix (26, 33). The analyst was trained by an experienced video analyst and researcher (AJG), who also conducted spot checks on all of the variables that were completed for the first season (i.e., 2018 NRLW season). The coding matrix comprised 36 categorical variables, the majority of which described characteristics of the tackle but also included pre-tackle characteristics (Appendix: Supplementary Table S1). The coding matrix was developed from the templates used in professional men's rugby union and previous work in men's rugby league (17, 26, 33, 34). A minimum of two camera view videos (25 fps) were available for each tackle, in normal speed and in slow motion. Every tackle event was coded across the three seasons of the NRLW competition. Tackles that did not result in an HIE (*n* = 6,318) formed a control sample that was used to calculate the frequency of each tackle characteristic in regular match play. This enabled calculation of the propensity of a given tackle scenario to cause an HIE In injuries per 1,000 tackles of each type and incidence, calculated as HIEs per 1,000 match hours.

A tackle event was defined as “any event where one or more tacklers attempted to stop or impede the ball carrier whether or not the ball carrier was brought to ground” (34). A ball carrier was defined as a player in possession of the ball when tackled by a tackler, including instances where the ball carrier offloaded the ball in the process of being tackled. Tacklers were defined as players attempting to impede the progress or divest an opponent in possession of the ball regardless of the outcome (e.g., incomplete tackles were included, defined as those where the defender made initial contact but missed the tackle due to physical contact with the ball carrier) (35).

In line with previous work in rugby union (27) and rugby league (26), this study focused specifically on the tackler and ball carrier's actions, reporting the body positions of the tackler and ball carrier, head contact during the tackle and the ball carrier's evasion methods. In accordance with Hopkinson and colleagues' (35) video analysis framework for the rugby league tackle, we considered variables from five out of the six “phases of the tackle” (i.e., the tackle event, defensive start point, pre-contact, initial contact, and post-contact, but we did not code any variable associated with the play-the-ball). In terms of tackler position, trunk posture has been defined as either upright (no flexion), bent at the waist (>60°) (36) or bent at the knees (>60° knee flexion), with a relatively upright torso. If a player was upright but had a significant bend in the knees (>60°), this was coded as bent at the knees. However, if the player's trunk was in greater flexion (>60°) than at the knees, this was coded as bent at the waist. Each of these components are summarised in categories for coded variables (see Supplementary File 1).

For the 2018–2020 seasons, two levels of in-game injury surveillance existed: (a) the sideline injury surveillance system via an independent medical bunker where spotters watched all matches with the specific objective of looking for signs of a concussion and (b) the club doctor. An HIE was defined as a clear head impact sustained by a player, which was monitored by sideline medical staff or the matchday video reviewer for potential follow-up in the form of an HIA. An HIA was identified as an HIE that necessitated a temporary exchange of a player with a suspected concussion for an off-field evaluation or the permanent removal of a player from the game with a confirmed concussion, as per the NRL concussion recognition and management process (18). Therefore, HIEs and HIAs differed in terms of the management approach adopted once a head impact was witnessed or via video review during the match. As described, for the analysis conducted in this study, HIEs (*n* = 83) have been used to ensure greater statistical power, given the low number of HIAs reported across the three seasons. Although HIAs do have an influence on game play, due to the temporary exchange or permanent removal of the player from the match, there were too few HIAs (*n* = 18) and medically diagnosed concussions (*n* = 6) to sufficiently power those analyses.

### 2.3. Data analysis

All analyses were conducted using IBM SPSS Statistics for Windows, version 24 (IBM Corp., Armonk, N.Y., USA). The event risk or propensity, in HIEs per 1,000 tackles for each tackle characteristic, was calculated by dividing the number of HIEs occurring from that tackle characteristic by the total number of occurrences of that tackle characteristic (obtained from the control cohort) and multiplying by one thousand. The incidence of HIEs was calculated as the number of HIEs per 1,000 hours of match play, and the period in matches per HIE for each tackle characteristic calculated. All results are presented as means and 95% confidence intervals (CIs). The probability of each tackle characteristic being associated with a player sustaining an HIE was assessed using Poisson regression with a log link function, using exposure to the characteristic as the offset variable to compare predictor/independent variables. Incident rate ratios (IRRs) were calculated to compare the propensity of two events by expressing the calculated HIE propensity relative to one another; 95% CIs were used based on the Poisson distribution and a difference was deemed to be significant if the 95% limits did not overlap (33).

## 3. Results

### 3.1. Overall summary

All tackles during the 3 years of the NRLW (2018–2020) competition were coded. Eighty-three HIEs were identified, of which 18 were HIAs, and 6 were medically diagnosed concussions. The control cohort comprised the 6,318 non HIE-tackles. Overall HIE tackle propensity was 12.9 (95% CI: 10.4–16.0) HIEs per 1,000 tackles, with one HIE in a tackle every 30 min (95% CI: 3.9–6.0). Forty-two and 39 HIEs occurred to tacklers and ball carriers, respectively. The remaining two HIEs occurred in open play, i.e., off the ball contact. Tackler HIE propensity was 6.6 (95% CI: 4.9–8.9) per 1,000 tackles, similar to that observed for ball carriers (6.1, 95% CI: 4.5–8.4) per 1,000 tackles.

Of the 18 HIAs, tacklers experienced 8 HIAs with a propensity of 1.3 (95% CI: 0.6–2.5) per 1,000 tackles compared to ball carriers (*n* = 10), with a propensity of 1.6 (95% CI: 0.8–2.9) per 1,000 tackles. Overall incidence of HIAs was 33 HIAs per 1,000 match hours. Differences between tackler and ball carrier HIA propensity were not significant.

The medically diagnosed concussions (*n* = 6), diagnosed as part of the three stage HIA process from the match to 2 days post match, had a propensity of 0.94 (95% CI: 0.4–2.1) per 1,000 tackles. There was one concussion every 3.5 matches, an incidence of 11 concussions per 1,000 match hours.

### 3.2. Interaction of tackler and ball carrier body positions

**Figure 1:**
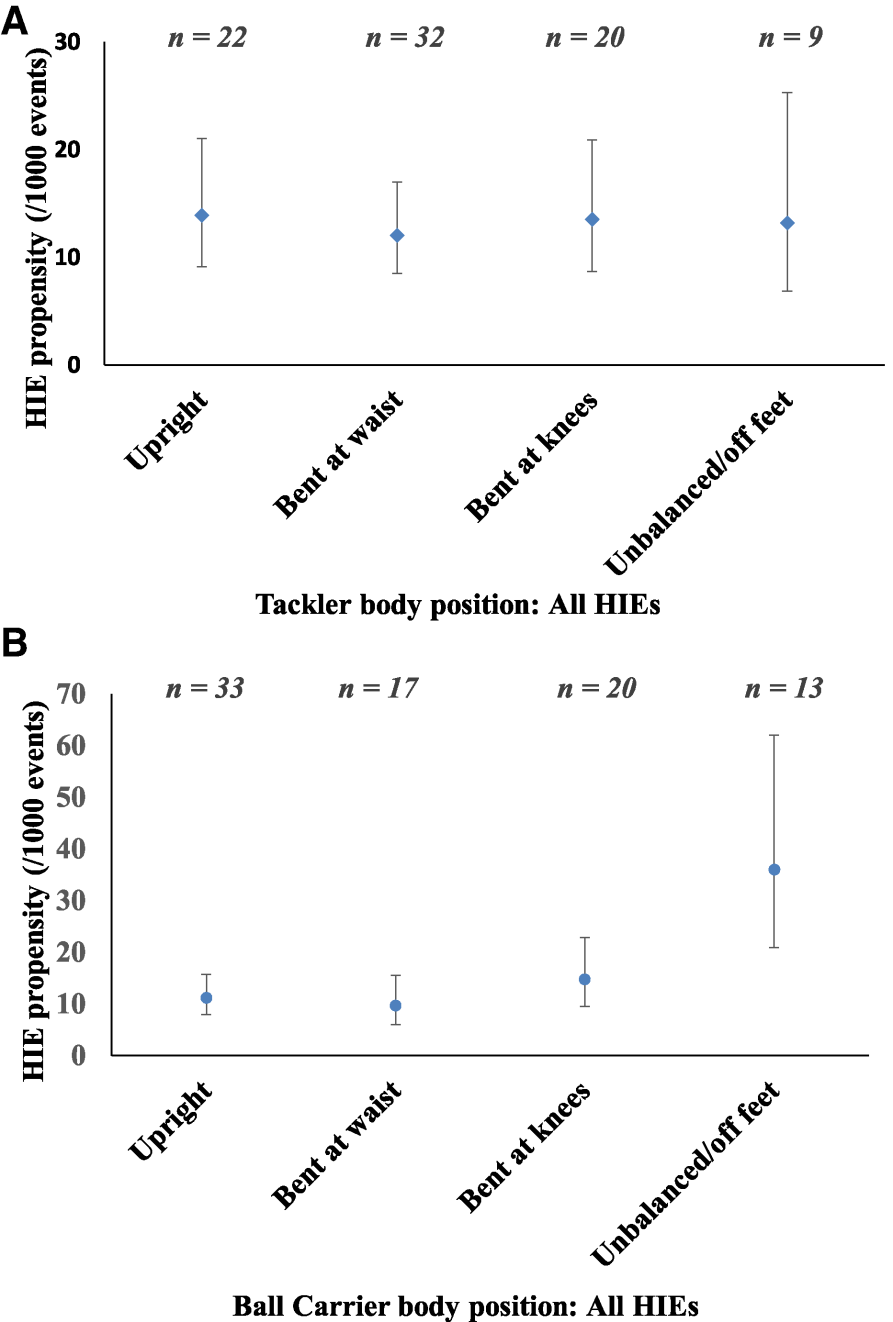
Tackler (**A**) and ball carrier (**B**) body position for head impact events (HIEs) to either player.

HIE propensity as a function of player body position is shown in Figure 1. Overall HIE propensity was similar for tacklers who were upright (13.9, 95% CI: 9.1–21.1 per 1,000 tackles), bent at the waist (12.0, 95% CI: 8.5–17.0 per 1,000 tackles), bent at the knees (13.5, 95% CI: 8.7–20.9 per 1,000 tackles), or unbalanced (13.2, 95% CI: 6.9–25.3) (Figure 1A). Upright tacklers were observed in 32% of all tackle HIEs and caused a HIE to either player every one match (95% CI: 0.6–1.5 per 1,000 tackles), an incidence of 41 HIEs per 1,000 match hours.

Considering ball carrier body position, overall HIE propensity was significantly greater when the ball carriers were unbalanced/ off their feet (36.0, 95% CI: 20.9–62.0 per 1,000 tackles), compared to upright (11.2, 95% CI: 7.8–15.8 per 1,000 tackles) and bent at the waist (9.7, 95% CI: 6.0–15.6 per 1,000 tackles), and similar to when the ball carriers were bent at the knees (14.8; 95% CI: 9.5–22.9 per 1,000 tackles; Figure 1B). Unbalanced/ off their feet ball carriers were observed in 15% of all tackle HIEs, with an incidence of 24 HIEs per 1,000 match hours.

The risk of an HIE to the tackler and ball carrier as a function of each player's body position were considered separately. No differences were found for tackler HIE propensity for different tackler body positions, nor for ball carrier HIE propensity, for different ball carrier body positions.

### 3.3. Location of body contact

Tackles with proximity of the tackler's head to the ball carrier's head or neck resulted in the highest HIE propensity of 287.2 per 1,000 tackles (95% CI: 197.0–418.9). This was significantly greater than tackles involving contact or proximity with the lower leg, upper trunk, lower trunk, and mid trunk (*p* < 0.05).

**Figure 2:**
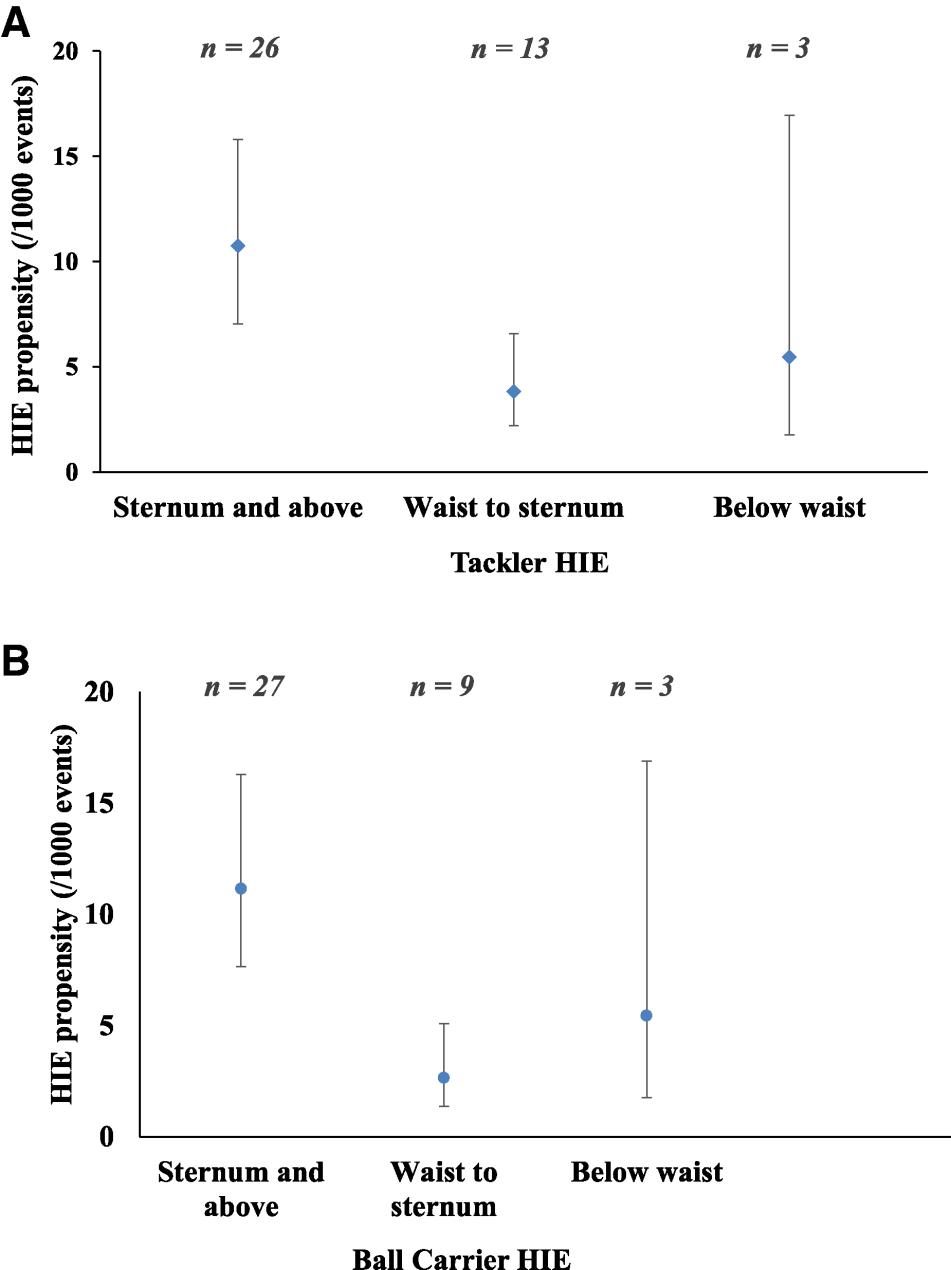
Propensity for head impact events (HIEs) based on head proximity location when (**A**) the tackler had an HIE and (**B**) the ball carrier had an HIE.

When grouped into tackles involving high contact (tackler's head was in close proximity to the ball carrier's sternum or above), medium contact (tackler's head between the ball carrier's sternum to waist), and low contact (tackler's head below the ball carrier's waist), tackler HIE propensity (Figure 2A) was 2.8 times greater for high contact than for medium contact (10.8, 95% CI: 7.3–15.8) per 1,000 tackles for high contact vs. 3.8 (95% CI: 2.2–6.6) per 1,000 tackles for medium contact. There was no significant difference between high and low, or medium and low contacts, with HIE propensity for low contacts at 5.5 (95% CI: 1.8–17.0) per 1,000 tackles.

Propensity for HIEs to the ball carrier (Figure 2B) was significantly higher when the tackler's head was above the ball carrier's sternum (11.2 per 1,000 tackles, 95% CI: 7.7–16.3), compared to when the head was between the waist and sternum (2.7 per 1,000 tackles, 95% CI: 1.4–5.1, IRR high vs. medium = 4.2). However, HIE propensity was not different when the tackler's head was in proximity to the ball carrier's waist and below (5.5 per 1,000 tackles, 95% CI: 1.8–16.9).

### 3.4. Head contact proximity and HIE propensity

For location of body contact, the HIE propensity and incidence are shown in Table 1. Tackler and ball carrier HIEs are considered separately. The highest propensity for tackler HIEs occurred with proximity between the tackler's and ball carrier's heads (214.3 per 1,000 tackles, 95% CI: 129.2–355.5), accounting for 36% of tackler HIEs. This was followed by proximity to the ball carrier's elbow (117.7 per 1,000 tackles, 95% CI: 29.4–470.4). HIEs from these impacts, however, were rare, accounting for 5% of tackler HIEs. The lowest propensity for tackler HIEs (7%) occurred with the tackler's head close to the ball carrier's arm, defined as point of contact between the shoulder and elbow (2.7 per 1,000 tackles, 95% CI: 0.8–8.2). However, contact with the ball carrier's torso had a lower propensity for tackler HIEs (4.0 per 1,000 tackles, 95% CI: 2.2–7.2, Table 1). The highest incidence for tackler HIEs was found in head-to-head impacts (27.5 HIEs per 1,000 match hours), followed by contact with the ball carrier's torso (20.1 HIEs per 1,000 hours).

**Table 1:** HIEs as a function of head contact proximity with the opponent's body part.

	HIEs (*n*)	Propensity (HIEs per 1,000 events)	95% CI	Incidence (events per 1,000 hours)
**Tackler HIEs as a function of tackler's head contact with ball carrier**				
Head	15	214.3	129.2–355.5	27.5
Elbow	2	117.7	29.4–470.4	3.7
Forearm	1	15.6	2.2–110.9	1.8
Hip	3	8.6	2.8–26.9	5.5
Thigh	2	4.9	1.2–19.8	3.7
Torso	11	4.0	2.2–7.2	20.1
Back	2	3.7	0.9–14.7	3.7
Shoulder	3	3.5	1.1–10.7	5.5
Arm	3	2.7	0.8–8.2	5.5
Total	42	6.6	4.9–8.9	76.9
**Ball carrier HIEs as a function of ball carrier's head contact with tackler**				
Ground	1	1,000.0	140.9–7,099.3	1.8
Head	10	526.3	283.2–978.2	18.3
Knee	1	250.0	35.2–1,774.8	1.8
Torso	7	5.1	2.5–10.7	12.8
Arm	10	4.7	2.5–8.7	18.3
Shoulder	10	4.7	2.5–8.7	18.3
Total	39	6.1	4.5–8.3	71.4

HIE, head impact event; CI, confidence interval.

The propensity for ball carrier HIEs was greatest when the ball carrier's head made contact with the ground, however, this was a rare event, accounting for only 2% of all ball carrier HIEs (1,000 per 1,000 tackles, 95% CI: 140.9–7,099.3). Head to head proximity with the tackler accounted for 25% of ball carrier HIEs, with a propensity of 526.3 per 1,000 tackles (95% CI: 283.2–978.2), followed by contact with the tackler's knee (250.0 per 1,000 tackles, 95% CI: 35.2–1774.8) and was lowest for contact with the tackler's arm and shoulder (4.7 per 1,000 tackles, 95% CI: 2.5–8.7). The highest incidence for ball carrier HIEs were contact with the tackler's head, arm, and shoulder, however, although head-to-head contact had a high propensity to cause ball carrier HIEs, contact with the arm and shoulder, had a relatively low propensity (4.7 per 1,000 tackles, 95% CI: 2.5–8.7, Table 1) for ball carrier HIEs.

## 4. Discussion

There is a paucity of literature examining mechanisms for head injuries in women athletes (10, 37–39). This study is the first to examine the propensity, incidence, and mechanism of HIEs, in Australian women's rugby league, during the first three seasons of the NRLW competition (2018–2020). We found that HIEs in the NRLW occurred at a propensity of 12.93 per 1,000 tackles, with an incidence of 152 tackle-related HIEs per 1,000 match hours. HIA propensity was lower than HIE propensity, at 2.80 per 1,000 tackles, equating to 33 HIAs per 1,000 match hours. The concussion propensity was 0.94 per 1,000 tackles, with an incidence of 11 concussions per 1,000 match hours. This is lower than in the NRL, where concussion incidence has been reported at 14.7 concussions per 1,000 match hours (2). However, because there were only six diagnosed concussions across the three seasons (i.e., 7 games per season), comparisons with the men's game (i.e., 24 games per team, per season) must be made with caution.

Our study's first important finding was that in the NRLW, tacklers and ball carriers were equally likely to sustain an HIE. Of the 83 HIEs, tacklers sustained 51% and ball carriers 47%, with the remaining 2% being off the ball contact. This differs from the body of research carried out in the men's NRL competition, where the tackler was more likely to experience an HIA during a tackle compared with the ball carrier (15, 16, 18, 26). This is a difference that may be, in part, the result of our identification of HIEs rather than HIAs, which was necessary due to the low number of HIAs (*n* = 18) reported across the three seasons in the women's game. It may be that tacklers and ball-carriers are at equal risk of experiencing head impact events, as we find for women, but that the likelihood that such a head impact will cause a clinical outcome (head injury or concussion) is greater in tacklers. This cannot be ascertained in the present study, but may be a focus of future research with a larger sample of athletes with concussion.

In terms of head impacts, it is possible that NRLW players are less experienced than men in the physical contact that is inherent in a tackle (8), and that the ball carriers may not be accustomed to being tackled, particularly when upright, making them more likely to experience HIEs than their male counterparts. This may be compounded by the technique and height of the tackler, whose head may more often be in proximity with the ball carrier's head, leading to a greater number of head-to-head impacts. This has been proposed in women's rugby union, where tackle technique is influenced by playing experience (40). In addition, due to inherent differences in neck strength and girth, women may not be able to efficiently dissipate the forces imparted in a tackle in the same way as men (41–44). Further research needs to elucidate whether increases in neck strength are associated with different clinical outcomes after head impacts in women (45, 46) and/or the ability to control the head during the forces imparted in the tackle.

NRLW players, along with the other football codes have attracted women from other sports, with reduced access to training, facilities, and coaching (10, 26). Furthermore, for the inaugural season (2018), players were contracted at short notice, for an overall average contract length of just 8 weeks (47). Players had a mean age of 27 years (range 18–42) across the first three seasons, which meant that some had to balance full or part-time employment, study, and caring for children while playing elite-level rugby league in an intense, short period of time (47). These women are unlikely to follow the same athletic preparation periodization as the men playing in the NRL competition, who were provided the necessary support structures to access a professional career in the NRL (47, 48). NRLW players often have not had the same viable career opportunities to pursue, which may have compromised their preparation and training in ways that have been linked with a higher injury incidence. This lack of experience, opportunity, and inherent inequalities in resourcing have been linked with a higher incidence of injuries (6–9).

Our second important finding was that risk of a head impact created by the tackler and ball carrier body position interactions differed to the risks of head injuries in men's rugby league and rugby union. Although the variables of interest are not the same (NRLW HIEs evaluate whether the head has been contacted, and the men's NRL and rugby union studies examined clinical outcomes following head impact) we compared the findings. In men's rugby league (17) and rugby union (28, 33), upright tacklers were significantly more likely to cause an HIA to either player than bent tacklers. Upright tackles in the NRL resulted in a higher proportion of head-to-head contact (26). We found the four observed tackler and ball carrier body positions resulted in a similar propensity for HIEs, which may indicate a different mechanism for HIEs for the NRLW players compared to HIAs and concussions in men's play, and which may reveal differences in tackle execution between women and men. It is possible that the women may choose to tackle differently from the men, to avoid breast tissue contact injuries (49), placing their heads in more upright or very low positions, to avoid upper-torso contact. This is of relevance because previous research has shown that tackles aimed at the mid-torso level are considered the safest zone in men (50, 51). Research from other sports such as soccer has revealed different concussion mechanisms for men and women, with concussions in men resulting from contact with another player, whereas in women, contact with an object or equipment is the most common mechanism for a concussion (52, 53).

Similarly, in university rugby union players, 45% of head impacts for women ball carriers were from head contact with the ground, compared with 57% of tacklers' heads contacting a hard body part of the opponent (54). For male tacklers and ball carriers, direct or indirect contact with another player had the greatest propensity for head injury (44). Although, our study did not find a body position significantly related to head impact risk, 33% of HIEs were sustained when the ball carrier was either unbalanced or off their feet (i.e., falling or diving). To reduce head impact risk in NRLW players, both tacklers and ball carriers may require tackle re-education, in terms of how the tackler performs the tackle and how the ball carrier engages in contact during the tackle. Current training strategies for women athletes have been derived from male data (40) and although the rules of rugby league may be similar for both sexes, athlete considerations in women need to be reflected in tackle technique and proficiency, size of the football as well as their dynamic balance and ability to dive or fall safely. A panel of experts identified poor tackle technique as the greatest risk factor for injury, specifically, in women's rugby league players, and was also the most feasible to change (38).

Third, with respect to head contact location or proximity, the highest propensity for HIEs occurred when the tackle height was above the sternum, with tackles to the head and neck resulting in the greatest propensity for head impact (Figure 2). These high contact tackles were significantly more likely to result in HIEs than tackles where contact occurs below the sternum. This is a finding similar to that observed in men's rugby union and rugby league (1, 17, 26–28, 50). In our study, tacklers were more likely to experience head-to-head and head-to-elbow impacts, while ball carriers more likely to have head-to-head impacts, head-to-knee impacts, or contact with the ground. In combination, this analysis suggests that when tackles are either too high (sternum and above) or too low (below the waist), the risk of head impacts is greater than when tackles are executed at between the sternum and waist. Given that tackling is a highly technical skill, tackle technique education and retraining to reduce head impact events, and potentially concussion incidence, may be warranted.

It is important to emphasize that we have focused on HIEs and not HIAs or concussions, and this makes direct comparisons with previous studies in men speculative. A subset of these head impacts would require removal of a player from the field for screening, and of these, concussions are a very small subset. We do not thus assess susceptibility to clinical injury risk, but rather likelihood of a head impact as a function of different tackle behaviors. It is possible that the risk factors for injury events differ from risk factors for impact events, by virtue of a different severity for the former. However, assessing head impacts remains important, because it provides stakeholders with a preliminary understanding of HIE risk within the emerging professional competition of the NRLW, under the premise that any reduction to HIE risk might also reduce injury outcomes, as well as purported theorized effects of cumulative exposure to head impacts. The relationship between head impacts and head injuries should be explored in future research.

As our results suggest that the tackler and ball carrier are equally likely to experience an HIE in women's rugby league, tackle technique and proficiency may have a role to play, as tacklers are primarily responsible for increasing head impact risk through their actions, which may then lead to head injury risk (55, 56). The ball carrier may also act to increase head impact risk, and so both players should be considered, but our findings suggest that modification to tackle height, as has been trialed in men's rugby union (57) in an attempt to reduce head injury risk, may also be warranted in women's rugby league. Furthermore, women may benefit from physical preparation strategies that prepare them for contact, in particular the ground contact that occurs when being tackled, and when diving for a loose ball that is either spilled on the ground or in the air.

### 4.1. Limitations

There a number of limitations associated with this study. Coding and evaluation of the tackle variables include elements of subjectivity. All events were coded by a single analyst, and the subjective nature represents a potential source of inaccuracy. When analyzing tackle characteristics, the nature of the tackle as a dynamic, multi-faceted event, occurring in real-time must be considered. Although we coded 36 individual variables that considered tackler and ball carrier interactions, head contact, and evasion methods, it is important to appreciate that tackle characteristics do interact with each other.

Second, due to the short seasons and relatively low number of games (*n* = 21), several tackle variables rarely occurred, leading to sparse data that should be interpreted with caution. This limitation in sample size also affects the analysis, because we cannot explore how HIE incidence and propensity may have differed between seasons, or between playing positions on the field, because any division of our data into smaller groups for this analysis means we would be statistically underpowered. However, these are important considerations for future research, which should identify whether HIE risk is changing over time, as well as which players are most at risk, and whether the HIE risk factors are similar to those that cause clinical outcomes such as HIAs and concussions.

Further research examining larger samples of women's rugby league players with quantification of head exposure risk via a combination of video analysis and instrumented mouthguard technology may be beneficial (58) to better inform our understanding of tackle collision risk. Future studies should also consider the role that level of experience, player position, and game demands play in injury risk.

## 5. Conclusions

Our study provides the first description of head impacts occurring in the first three seasons of the NRLW competition. Our preliminary results show that professional women's rugby league tacklers and ball carriers are at equal risk of sustaining a tackle-related HIE. Head-to-head proximity and tackles above the sternum carry the greatest risk of HIEs in the NRLW. Injury prevention initiatives for women may need to consider both the tackler's execution and ball carrier's engagement, in order to influence risk. Current player welfare strategies in women's sport are still informed by male derived data and this warrants caution in transferring evidence-based men's injury reduction strategies to the women's game. Therefore, large prospective studies focusing on the women's game would better inform head injury risk and prevention strategies.

## Key points

During a tackle, in the NRLW, tacklers and ball carriers are equally at risk of sustaining a head impact. Head-to-head proximity and legal tackles at and above the sternum carry the greatest risk of HIEs in the NRLW. Consistent with men's rugby league, injury prevention initiatives aimed at reducing tackle height might reduce the HIE risk in women. Furthermore, in women, both the tackler's execution and ball carrier's engagement need to be considered from a technique re-education perspective to reduce risk.

## Data Availability

The datasets presented in this article are not readily available because of privacy considerations. The original contributions presented in the study are included in the article, further inquiries can be directed to the corresponding author. The statistical code, syntax, output, and analyses are available to qualified researchers upon request. Requests to access the datasets should be directed to andrew.gardner@sydney.edu.au.
